# Low-Dose BPA Exposure Alters the Mesenchymal and Epithelial Transcriptomes of the Mouse Fetal Mammary Gland

**DOI:** 10.1371/journal.pone.0063902

**Published:** 2013-05-21

**Authors:** Perinaaz R. Wadia, Nicolas J. Cabaton, Michael D. Borrero, Beverly S. Rubin, Carlos Sonnenschein, Toshi Shioda, Ana M. Soto

**Affiliations:** 1 Department of Anatomy and Cellular Biology, Tufts University School of Medicine, Boston, Massachusetts, United States of America; 2 Center for Cancer Research, Massachusetts General Hospital, Charlestown, Massachusetts, United States of America; Deakin School of Medicine, Australia

## Abstract

Exposure of rodent fetuses to low doses of the endocrine disruptor bisphenol A (BPA) causes subtle morphological changes in the prenatal mammary gland and results in pre-cancerous and cancerous lesions during adulthood. To examine whether the BPA-induced morphological alterations of the fetal mouse mammary glands are a) associated with changes in mRNA expression reflecting estrogenic actions and/or b) dependent on the estrogen receptor α (ERα), we compared the transcriptomal effects of BPA and the steroidal estrogen ethinylestradiol (EE2) on fetal mammary tissues of wild type and ERα knock-out mice. Mammary glands from fetuses of dams exposed to vehicle, 250 ng BPA/kg BW/d or 10 ng EE2/kg BW/d from embryonic day (E) 8 were harvested at E19. Transcriptomal analyses on the ductal epithelium and periductal stroma revealed altered expression of genes involved in the focal adhesion and adipogenesis pathways in the BPA-exposed stroma while genes regulating the apoptosis pathway changed their expression in the BPA-exposed epithelium. These changes in gene expression correlated with previously reported histological changes in matrix organization, adipogenesis, and lumen formation resulting in enhanced maturation of the fat-pad and delayed lumen formation in the epithelium of BPA-exposed fetal mammary glands. Overall similarities in the transcriptomal effects of BPA and EE2 were more pronounced in the epithelium, than in the stroma. In addition, the effects of BPA and EE2 on the expression of various genes involved in mammary stromal-epithelial interactions were suppressed in the absence of ERα. These observations support a model whereby BPA and EE2 act directly on the stroma, which expresses ERα, ERβ and GPR30 in fetal mammary glands, and that the stroma, in turn, affects gene expression in the epithelium, where ERα and ERβ are below the level of detection at this stage of development.

## Introduction

The endocrine disruptor bisphenol A (BPA) is ubiquitous in the environment. Its estrogenic activity was first identified in the 1930s [1]. Since the 1950s, BPA has been used in the manufacture of polycarbonate plastics, epoxy resins, dental sealants, as well as numerous other products [2], [3]. Measurable levels of BPA have been detected in human neonatal and adult serum, amniotic fluid, placenta, milk and urine [4]. Higher BPA levels in urine have been found in children when compared with adults [5]. Blood sampling revealed that internal exposures to the active, un-conjugated BPA in human serum are in the range of 0.5–10 ng/mL, with most studies suggesting an average internal exposure of approximately 1–3 ng/mL [6].

*In vitro* studies involving nuclear estrogen receptors have revealed that BPA is significantly less potent than estradiol-17β; for this reason, some considered it a “weak” estrogen (reviewed in [2]). However, studies involving fetal and perinatal exposure to low doses of BPA in rodents have documented persistent alterations in the structure and function of estrogen target tissues such as the hypothalamus [7], ovaries, vagina, uterus [8] and mammary glands [9]–[12]. At embryonic day 18 (E18), mammary glands of the BPA-exposed CD-1 mouse fetuses revealed accelerated differentiation of the fat pad and altered collagen localization in the mesenchyme, while a decrease in cell size, delayed lumen formation, and increased ductal area were observed in the epithelium [13]. In mice perinatally exposed to BPA, a delay in ductal invasion of the mammary stroma was observed at puberty, and increased lateral branching and terminal ends were observed at adulthood [9], [10]. The mammary glands of mice exposed perinatally to a low dose of BPA also exhibited a heightened response to estradiol during adult life [14] and developed intraductal hyperplasias, which are considered pre-neoplastic lesions [15]. Similarly, in the rat model, prenatal exposure to BPA resulted in the development of intraductal hyperplasias and carcinomas *in situ* [16]. Exposure to BPA during development was also linked to increased sensitivity of the rat mammary gland to chemical carcinogens [17], [18]. These studies clearly indicate that the fetal mammary gland is a target of BPA and that its effects exacerbate at puberty and beyond, long after exposure has ended.

In this study we determined whether transcriptional changes occurred in E19 peri-ductal stroma and epithelium of BPA-exposed fetal mammary glands at a dose which had previously been shown to result in morphological alterations at this time-point [13]. The analysis of these two compartments of the mammary gland was important as mammogenesis and carcinogenesis are mediated by stromal-epithelial interactions [19]. Transcriptional effects of BPA were compared with those of a potent steroidal estrogen, ethinylestradiol (EE2), to obtain insights into whether the biological effects of BPA were explained by its estrogenic activity. We also used ERα-null [ERα(−/−)] mice [20] to determine roles of estrogen receptor α (ERα) in the transcriptional actions of BPA and EE2. Our data indicate that BPA and EE2 differentially affect the transcriptomes of the fetal mammary epithelium and peri-ductal stroma, with an implication that these estrogenic compounds act directly on the stromal cells, which in turn affect gene expression in the epithelial cells.

## Methods

### Animals

Mice heterozygous for the ERα gene [ERα(+/−)] of C57BL/6 background were generously provided by Dr. Pierre Chambon [20]. Mice were maintained in temperature- and light-controlled (14-hr light, 10-hr dark cycle) conditions at the Tufts University School of Medicine animal facility. All animal procedures were approved by the Tufts University and Tufts Medical Center Institutional Animal Care and Use Committee in accordance with the Guide for Care and Use of Laboratory Animals. Cages and bedding tested negligible for estrogenicity by the E-SCREEN assay [21]; water was supplied from glass bottles only. Food (2018 Rodent Diet, Harlan Teklad; Indianapolis, IN) was supplied *ad libitum*; estrogenicity of feed was measured at <20 pmol of estrogen equivalents per gram [21].

Sexually mature ERα(+/−) male and ERα(+/−) female mice were mated. The morning of vaginal plug observation was designated E1. Mice were weighed and implanted, on the evening of E8, with Alzet osmotic pumps (Alza Corp., Palo Alto, CA) designed to deliver either 50% dimethylsulfoxide (vehicle control) or 250 ng BPA/kg BW/d or 10 ng EE2/kg BW/d (positive control for estrogenic action) dissolved in vehicle. The actual dose delivered to pregnant dams would be expected to decrease as pregnancy progressed due to increasing weight of the fetuses (the pregnant dams increased their body weight by 37% on average between E8 and E19). The treatment lasted until the mice were sacrificed on E19. Pregnant mice were injected with a sub-lethal dose of ketamine and xylazine and the fetuses were delivered by caesarean section and decapitated. The genotypes of fetuses were determined from tail snips [20], and only female ERα(+/+) and ERα(−/−) fetuses were used in the study. Female fetuses were identified by anogenital distance and confirmed by the presence of ovaries and uteri during dissection. One typical fetus was selected from each litter and subjected to analyses. Similarly, from each treated litter, only one fetus of each genotype [ERα(+/+) and ERα(−/−)] was sampled. This study was conducted in C57BL/6 mice, because this strain was found to be sensitive to BPA [14] and also because the ERα(−/−) mice, used in this study, were of this strain. Previous morphometric analyses of fetal mammary glands exposed to BPA had been conducted in the CD-1 mouse sacrificed at E18 [13]. A comparison of the development of fetal mammary glands of these two mouse strains demonstrated that the C57BL/6 at E19 is most comparable to the E18 stage in the CD1 mouse (Figure S1 and Table S1).

### Tissue Collection and Preparation

The fourth inguinal mammary glands of female fetuses (from three independent litters per treatment group for both ERα(+/+) and ERα(−/−) mice) were isolated with the aid of a stereo-microscope (Zeiss New York, NY) under RNAse-free conditions as described previously [13]. The glands were spread onto porous specimen collection bags and then placed in vials containing RNAlater (Invitrogen, Carlsbad, CA), for mRNA expression studies or fixed in 4% paraformaldehyde in 0.1 M phosphate buffered saline (PBS) for 18–20 hours at room temperature for histological analyses. The glands preserved in RNAlater at 4°C for 2–5 days were embedded with Optimal Cutting Temperature (OCT) compound and only one mammary gland/fetus was sectioned on a cryostat (Microm HM560) (18 µm thickness). The paraformaldehyde-fixed tissues were embedded in paraffin and sectioned on a microtome (Leica RM2155) (5 µm thickness).

### Laser Capture Microdissection

One mammary gland from each pup was sectioned in its entirety and all frozen sections were stained with the Arcturus HistoGene Frozen Section Staining kit (Applied Biosystems, Carlsbad, CA) under RNase-free conditions using the manufacturer’s protocol. Only sections containing epithelial ducts, which amounted to 4–6 sections/mammary gland, were used further. The peri-ductal stroma, which corresponded to approximately 100 µm from the outer boundary of the epithelium, and the epithelial ducts were separately captured using the Arcturus PixCell IIe Laser Capture Microdissection System (Applied Biosystems, Carlsbad, CA) (Figure S2). All the epithelium from one mammary gland was pooled into one tube while all of the peri-ductal stroma was collected in a second tube for a single sample of each treatment and genotype.

### RNA Isolation and Microarrays

Total RNA was isolated from the laser-captured tissue specimens using the Arcturus PicoPure RNA isolation kit (Life Technologies Corporation, Carlsbad, CA) and amplified using the Ovation Pico WTA System (NuGEN Technologies, San Carlos, CA). Amplified cDNA (5 µg/sample) was labeled with biotin using the Encore Biotin Module (NuGEN Technologies) and then hybridized to the Mouse Genome 430 2.0 Array (Affymetrix Santa Clara, CA) according to the manufacturer’s instructions. The extremely limited amounts of the laser-captured RNA samples prevented direct assessments of their integrity. However, size distributions of the amplified cDNA species determined by Agilent Bioanalyzer (Agilent Technologies, Santa Clara, CA) did not show any significant signs of RNA degradation. The 3′/5′ signal intensity ratios of housekeeping genes detected by Affymetrix microarrays were not greater than 2.0, supporting the integrity of the RNA samples.

The.CEL files were generated by Affymetrix GCOS and subjected to the Robust Multiarray Averaging (RMA) method of normalization using Partek Genomics Suite (Partek Inc., St. Louis, MO). The normalized microarray signal intensities were log transformed, and differentially expressed genes were identified by one-way ANOVA (p<0.05) using Partek or by Welch’s *t*-test (two-sided, p<0.05; further filtered for genes whose expression changed by at least 2-fold) using GeneSifter Analysis Edition v3.7 (Geospiza, Inc., Seattle WA). Because applications of multiple testing corrections (Bonferroni and Benjamini-Hochberg) to these microarray data sets resulted in excessive suppression of discovery power for identification of differentially expressed genes, the subsequent analyses were performed with genes selected without such corrections. Two-dimensional hierarchical clustering was performed using Partek, and Gene Ontology analysis was performed using GeneSifter. The signal intensities of specific transcripts of interest determined by microarrays were compared to identify significant differences between the treatment groups (t-test, p<0.05 cutoff).

### Real Time qRT-PCR

SYBR Green quantitative RT-PCR was performed as previously described [9]. Forward and reverse PCR primers were as follows: *Krt8* (F: gagtctgggatgcagaacatgag; R: gtgcggctgaaagtgttgg), *Fabp4* (F: ctggtgcaggtgcagaagt; R: aatttccatccaggcctctt), *PPARγ* (F: aggccgagaaggagaagctgttg; R: tggccacctctttgctctgctc), and *RPL19* (Ribosomal protein L19) (F: atcgccaatgccaactcc; R: tcatccttctcatccaggtca).

RNA isolated from an intact E19 mammary gland section was included as a positive control calibrator. The cycle threshold values of the gene of interest for each sample were normalized to the housekeeping gene (*RPL19*) followed by the calculation of fold-change in gene expression with respect to the calibrator sample.

### Immunohistochemistry

Paraffin sections were treated with xylene to remove paraffin and rehydrated through a series of alcohols and PBS. For antigen retrieval, sections were exposed to pepsin (0.1% in 0.01 N HCl, pH2.0; Sigma-Aldrich Co., MO) for 50 minutes at 37°C. Sections were incubated overnight at 4°C in a humid chamber with primary antibody for the focal adhesion protein Tenascin (1∶150 dilution, Millipore Corp, Billerica MA). Biotinylated goat anti-rabbit IgG (1∶150 dilution, Zymed, San Francisco, CA) was applied to sections for 1 hour in a humidified chamber at room temperature. Slides were rinsed with PBS and detection of positive cells was accomplished using alkaline phosphatase (Vectastain ABC-AP kit; Vector, Burlingame, CA). Samples were counterstained with Harris’ hematoxylin, dehydrated and mounted in a permanent mounting medium.

## Results

**Figure 1:**
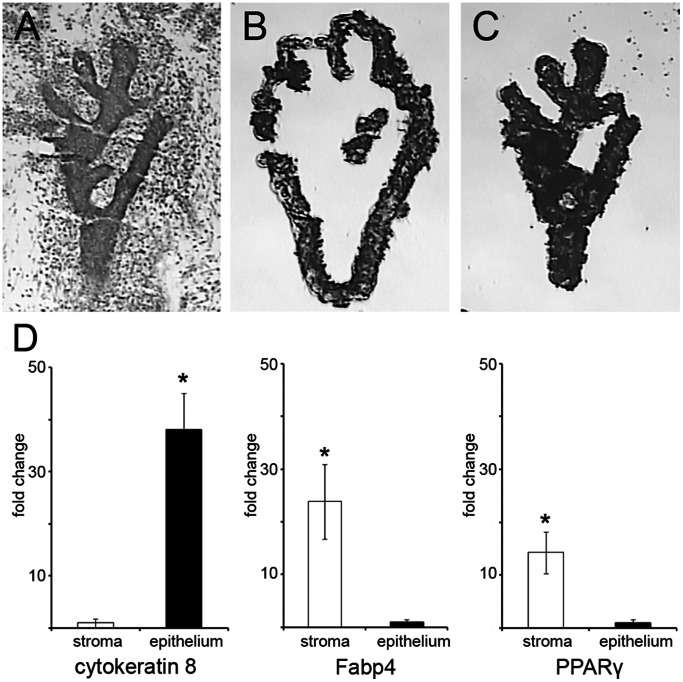
Isolation of the mammary epithelium and peri-ductal stroma. Laser capture microdissection of the epithelium and peri-ductal stroma of E19 mammary gland; (A) Frozen section stained with Histogene stain; (B) peri-ductal stroma surrounding the epithelium isolated on an HS LCM cap; (C) epithelium isolated on an HS LCM cap; (D) expression of genes specific to the peri-ductal stroma and epithelium assessed by Real-Time RT-qPCR. Data expressed as fold change relative to the tissue compartment with least expression (*p<0.05).

Mammary glands of E19 female fetuses, exposed to BPA (250 ng/kg BW/day), EE2 (10 ng/kg BW/day), or vehicle from E8 until E19, with ERα(−/−) and ERα(+/+) genotypes were harvested and the epithelium and peri-ductal stroma of these glands were separated by LCM. Accurate LCM capture of the epithelial and peri-ductal stromal compartments is shown in Figure 1A–C (and Figure S2). Expression of the mRNA transcripts for an epithelium-specific marker gene (*Krt8*, encoding cytokeratin 8) and the mammary stroma-specific marker genes (*Fabp4* and *PPARγ*, encoding fatty acid binding protein 4 and peroxisome proliferator activated receptor γ, respectively) demonstrated greater than 95% purity of each of these laser-captured compartments (Figure 1D).

### Transcriptional Effects of BPA and EE2 on Fetal Mouse Mammary Stroma and Epithelium

**Figure 2:**
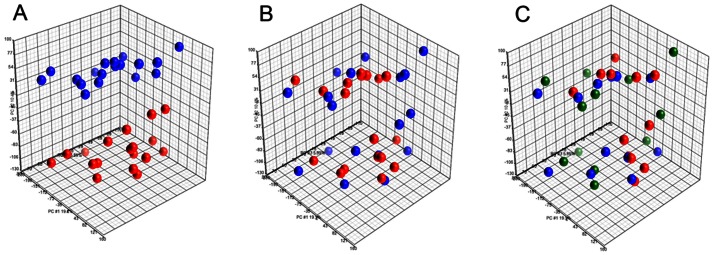
Principal Component Analysis of the global gene expression profile. Principal Component Analysis of the microarray data of E19 mammary glands indicating differences in global gene expression (A) between the epithelium (red) and peri-ductal stroma (blue); (B) between ERα(+/+) (blue) and ERα(−/−) mice (red) and (C) between the three treatment groups: BPA (red), EE2 (blue) and vehicle (green).

In order to determine transcriptional effects of fetal exposure to BPA and EE2 in the epithelial and stromal compartments of fetal mouse mammary glands, mRNA expression profiles of the LCM-captured tissues were examined by Affymetrix microarray. Principal component analysis (PCA) on their transcriptomal profiles revealed a clear separation of the epithelial and stromal compartments (Figure 2A), confirming the absence of significant cross contaminations between these two distinct compartments. As expected from the PCA which can identify only gross variability [22], [23], no significant transcriptomal changes caused by the deficiency of ERα (Figure 2B) or exposure to the estrogenic compounds were observed (Figure 2C). Likewise the morphological differences observed in the mammary glands at this age between the stroma and epithelium is obvious, while the difference due to hormone treatment is subtle.

**Figure 3:**
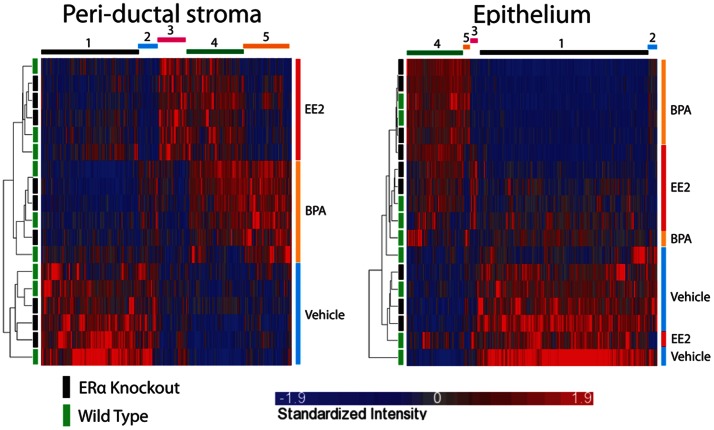
Hierarchical Clustering Analysis of the global gene expression profile. Effect of fetal exposure to BPA, EE2 and vehicle on the transcriptomal profiles of (A) peri-ductal stroma and (B) epithelium: hierarchical clustering analysis. Status for ERα is indicated on the left (Green: ERα(+/+), Black: ERα(−/−)) and treatment is indicated on the right of the heat maps. Unique gene clusters observed are shown on the top of the heat maps.

In the peri-ductal stroma, hierarchical clustering analysis on 2036 genes pre-selected by ANOVA for differential expression (p<0.05) between vehicle and BPA or vehicle and EE2 demonstrated clear separation of their mRNA expression profiles along the three exposure groups (Figure 3). In this heatmap, five distinct clusters of genes were recognized as shown in Table S2. Figure 3 also shows a heatmap of the same hierarchical clustering analysis on 5348 genes selected by ANOVA (p<0.05), expressed in the epithelial compartment. The size of the gene clusters representing differential transcriptional effects of BPA and EE2 (*i.e.,* sum of numbers of genes belonging to clusters 2, 3, and 5) were remarkably smaller in the epithelium compared with those of the peri-ductal compartment (459 of 5348 genes for the epithelial compartment *versus* 789 of 2036 genes for the stromal one; p<0.001-Fisher Exact Test). These results suggest that the transcriptional response of peri-ductal stromal cells partially distinguishes the two estrogenic agents (BPA and EE2) whereas the response to these agents by epithelial cells was largely identical. The absence of ERα did not significantly alter the global transcriptional responses to BPA and EE2 of either compartment. This result suggests that ERα may not play a major role as a mediator of the BPA effects observed during fetal life. However, these observations do not rule out involvement of ERα for the regulation of a small set of transcripts as shown below.

### BPA Effects on mRNA Expression of Genes in the Mammary Peri-ductal Stroma Correlates with Morphological Changes

**Figure 4:**
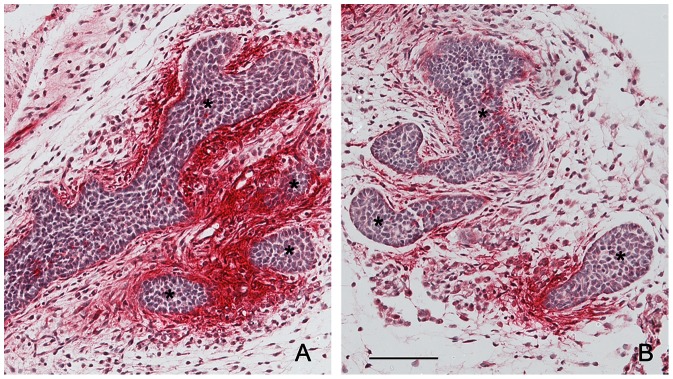
Localization of TnC in the fetal mammary gland. Immunohistochemical localization of TnC in (A) vehicle and (B) BPA-exposed mammary glands of ERα(+/+) mice at E19. Red alkaline phosphatase staining of TnC is observed in the peri-ductal stroma. (*epithelial ducts; scale bar = 100 µm).

Gene Ontology (GO) analysis on genes with altered mRNA expression following fetal exposure to BPA in the peri-ductal stroma of ERα(+/+) mice pointed to alterations in the focal adhesion pathway (Z-scores: up-regulation by BPA = 1.08, down-regulation by BPA = 2.3). Expression of the mRNA transcripts for genes encoding the extracellular matrix components, such as tenascin (*Tnc*), was significantly down-regulated along with other extracellular matrix components (Table 1) in the BPA-exposed mice. Agreeing with this observation made by mRNA microarray analysis, immunohistochemical examination demonstrated a significantly decreased expression of tenascin protein in the peri-ductal stroma of BPA-exposed mice (Figure 4). Additionally, the actin-binding protein filamin b (Flnb) was up-regulated by BPA exposure (signal intensity in arbitrary units were 31.8±4.2 and 445.6±245.3 for vehicle- and BPA-exposed mice, respectively). These results correlate with our previous findings showing profound changes in matrix organization in the peri-ductal stroma [24]. This in turn may alter the biomechanical properties of the cells within it which reorganize their cytoskeleton, as suggested by the expression of filamin b.

**Table 1:** Focal adhesion-pathway in the stroma.

				Signal Intensity				
Gene Name	Gene ID	Probe Set ID	Gene Identifier	Vehicle±SEM	BPA±SEM	Fold-change	p-value	Direction in BPA group
Tenascin C	Tnc	1416342_at	NM_011607	2130.30±179.92	998.56±178.57	2.13	0.0111	Down
Versican	Vcan	1447887_x_at	AV245031	1104.93±150.68	417.9±112.01	2.64	0.0248	Down
Asp6 mRNA for adipocyte-specific protein 6	Col5a3	1419703_at	NM_016919	858.20±59.42	427.36±60.53	2.01	0.0071	Down
Cyclin D1	Ccnd1	1417420_at	NM_007631	1028.56±107.10	508.73±113.99	2.02	0.0295	Down

Genes involved in the ontology category of the ‘focal adhesion’ pathway altered in the peri-ductal stroma of ERα(+/+) BPA mice compared to ERα(+/+) Vehicle mice. (The Affymetrix probe ID and gene identifier are mentioned for each transcript of interest).

Although the adipogenesis pathway did not appear to be altered in our microarray data, significant changes in mRNA expression of several genes involved in adipogenesis were detected. In the peri-ductal stroma of both ERα(+/+) and ERα(−/−) mice, significant increases in the expression of *PPARγ*, a master gene of adipogenesis, as well as other adipogenic genes such as low density lipoprotein receptor (*Ldlr*), G protein-coupled receptor 81 (*GPR81*), and *Fabp4* were observed in the BPA-exposed mammary glands (Table 2). This increased expression of genes involved in adipogenesis is consistent with our previous observations at E18 showing an increase in the number of adipocytes containing lipid droplets and expressing Fabp4, in the peri-ductal stroma of BPA-exposed mice [24].

**Table 2:** Adipogenesis in the stroma.

				Signal Intensity				
Gene Name	Gene ID	Probe Set ID	Gene Identifier	Vehicle±SEM	BPA±SEM	Fold-change	p-value	Direction in BPA group
Peroxisome proliferator-activated receptorgamma	Pparg	1420715_a_at	NM_001127330	618.64±124.17	1463.74±271.63	2.36	0.0323	Up
Fatty acid bindingprotein 4	Fabp4	1425809_at	NM_024406	8594.94±230.60	10814.46±604.64	1.26	0.0178	Up
Low densitylipoproteinreceptor	Ldlr	1459403_at	NM_010700	44.56±7.43	163.88±23.09	3.67	0.0048	Up
G protein-coupledreceptor 81	GPR81	1438411_at	NM_175520	57.47±18.26	279.68±51.38	4.87	0.0060	Up

Genes involved in adipogenesis, identified in the peri-ductal stroma of vehicle versus BPA-exposed mice. Data from ERα(+/+) and ERα(−/−) mice for both treatment groups was pooled for this analysis. (The Affymetrix probe ID and gene identifier are mentioned for each transcript of interest).

### BPA Effects on mRNA Expression of Genes in the Mammary Epithelium Correlates with Morphological Changes

In the epithelium, the GO category of genes regulating apoptosis was altered by BPA exposure in ERα(+/+) mice (Z-scores: up-regulation by BPA = 2.03, down-regulation by BPA = 1.88). The anti-apoptotic genes baculoviral IAP repeat-containing protein 2 (*Birc2*) and v-abl Abelson murine leukemia viral oncogene homolog 1 (*Abl1*) were significantly up-regulated in BPA-exposed mice (Table 3). These results are consistent with our previous observations of decreased apoptosis in the ductal cords of BPA-exposed mice, resulting in delayed lumen formation [24].

**Table 3:** Regulation of apoptosis-pathway in the epithelium.

				Signal Intensity				
Gene Name	Gene ID	Probe Set ID	Gene Identifier	Vehicle±SEM	BPA±SEM	Fold-change	p-value	Direction in BPA group
Baculoviral IAP repeat-containing 2, mRNA	Birc2	1418854_at	NM_007465	108.57±40.24	328.20±9.50	3.02	0.0270^a^	Up
Synovial apoptosis inhibitor 1, synoviolin (Syvn1), mRNA	Syvn1	1443609_s_at	C85322	71.63±7.67	196.60±31.94	2.74	0.0526	Up
V-abl Abelson murine leukemia oncogene 1, mRNA	Abl1	1423999_at	J02995	146.10±15.16	317.20±44.45	2.17	0.0492^a^	Up

Genes involved in the ontology category of ‘regulation of apoptosis’ altered in the epithelium of ERα(+/+) BPA mice compared to ERα(+/+) Vehicle mice. (‘a’ indicates statistical significance; the Affymetrix probe ID and gene identifier are mentioned for each transcript of interest).

### Expression of Hormone Receptors that Bind to BPA

BPA is known to bind to receptors other than ERs, albeit at significantly lower affinity. Messenger RNA transcripts for these receptors were expressed in the mammary peri-ductal stroma and epithelium (Table 4). Expression of ERα was observed only in the stroma of ERα(+/+) mice agreeing with a previous study [25], and was absent in the ERα(−/−) mice (Figure S3). ERβ and estrogen-related receptor gamma (*Esrrg*) are known to be activated by binding to BPA [26], but the mRNA transcripts for these genes were not detected in either the epithelium or the peri-ductal stroma of the fetal mammary glands. However, the expression of ERβ has been shown to occur in the whole mammary gland at E18 in CD1 mice [24] as well as in mammary glands of C57BL/6 mice that are cleared of epithelium (whole stroma) at E19 (unpublished data). ERβ expression is clearly absent in the epithelial ducts and may hence be expressed in the regions beyond the peri-ductal stroma that was examined in the current study. The G protein-coupled receptor 30 (*GPR30*), a recently discovered receptor for BPA and estrogen [26], [27], was expressed only in the peri-ductal stroma of fetal mammary glands of ERα(+/+) mice and was not detectable in ERα(−/−) mice. Androgen receptor (*Ar*) was expressed only in the stroma whereas thyroid hormone receptor α (*Thra*) and glucocorticoid receptor (*Nr3c1*) were expressed in both the peri-ductal stroma and epithelial compartments (Table 4).

**Table 4:** Expression of receptors that bind BPA.

Gene name	Gene ID	Expression in stroma		Expression in epithelium	
		WT	KO	WT	KO
Estrogen receptor alpha	Esr1	+	–	–	–
Estrogen receptor beta	Esr2	–	–	–	–
Estrogen-related receptor gamma	Esrrg	–	–	–	–
G protein-coupled receptor 30	Gper	+++	–	–	–
Glucocorticoid receptor/nuclear receptor subfamily 3, group C, member 1	Nr3c1	++	++	++	++
Thyroid hormone receptor alpha	Thra	+++	+++	++	+++
Thyroid hormone receptor beta	Thrb	–	–	–	–
Androgen receptor	Ar	+	+	–	–

Transcripts detected in epithelium and peri-ductal stroma of the mammary gland in ERα(+/+) and ERα(−/−) mice treated with vehicle. [Signal intensity in arbitrary units: +(100–200); ++(200–400); +++(400 and higher); – (below 100)].

### Transcripts Potentially Regulated by BPA and EE2 through ERα

First, we identified genes with mRNA expression affected at least 2-fold by exposure to either BPA or EE2 compared to vehicle. Next, we removed genes that showed comparable estrogen responsiveness (p<0.05) in the ERα(+/+) and the ERα(−/−) mice. When this procedure was applied to both epithelial and stromal compartments, a total of 266 genes passed these criteria (Table S3a-d), and five of them were known to be involved in mammary gland development (Table 5). The hepatocyte growth factor receptor/Met proto-oncogene (*Met*), implicated in branching of the mammary gland [28], was up-regulated in the ERα(+/+) epithelium following exposure to estrogens but not in the epithelium of the ERα(−/−) mice. Transglutaminase 2 (*Tgm2*), known to catalyze extracellular matrix cross-links [29], [30], was also up-regulated upon exposure to the estrogens in the epithelium. In contrast, zyxin (*Zyx*), which is involved in mechanical-force-dependent facilitation of actin polymerization at focal adhesions [31], was down-regulated in the estrogen-exposed epithelium. In the peri-ductal stroma, peptidyl-prolyl cis/trans isomerase (*Pin1*), a gene overexpressed in breast tumors [32], was up-regulated following exposure to the estrogens while the tripartite motif-containing 29 (*Trim29*), a gene suppressed in prostate and breast cancer [33], [34] was down-regulated in an ERα dependent manner.

**Table 5:** Estrogen-regulated transcripts dependent on ERα expression.

				WT			KO		
Name	Gene Symbol	Probe Set ID	Tissue/Expression	BPA/Veh	EE2/Veh	Highest ratio	BPA/Veh	EE2/Veh	Highestratio
Zyxin	Zyx	1417240_at	Epithelium/Down	1.5	2.6	2.6	0.7	0.8	0.8
Trans-glutaminase 2, C polypeptide	Tgm2	1417500_a_at	Epithelium/Up	2.4	3.0	3.0	0.7	0.7	0.7
Hepatocyte growth factorreceptor/Metproto-oncogene	Met	1434447_at	Epithelium/Up	1.7	2.9	2.9	1.1	0.9	1.1
Tripartite motif-containing 29	Trim29	1424162_at	Stroma/Down	4.0	4.0	4.0	0.6	0.9	0.9
Peptidyl-prolyl cis/trans isomerase	Pin1	1416228_at	Stroma/Up	1.5	2.1	2.1	1.0	0.6	1.0

Transcripts affected by estrogen treatment in the ERα(+/+) but not in ERα(−/−) mice. (The values in the table indicate the ratio of the average signal intensity for that gene. Only genes with an increase or decrease in expression ratio ≥2.0, between vehicle and at least one estrogen treatment, are listed. The Affymetrix probe ID and gene identifier are mentioned for each transcript of interest).

## Discussion

Previous studies in our lab, conducted in mice born to mothers dosed with 250 ng BPA/kg BW/d as in the present study, have shown an altered fetal mammary gland phenotype [13]. This dose, administered to their mothers is considered a ‘low dose’ as it is 200-times lower than the reference dose of 50 µg/kg BW/d defined by the US Environmental Protection Agency [35] which is the maximal acceptable oral dose of a toxic substance. In addition, the levels of total BPA in the serum of the pregnant mice administered this dose would be below the detectable limit of 0.3 ng/ml (BSR- personal communication). In order to determine whether the effects previously observed at this BPA dose are due to its estrogenic activity, we performed transcriptomal analyses on the stromal and epithelial compartments of the fetal mouse mammary gland during the period of BPA or EE2 exposure. Clustering analysis demonstrated significant similarities in the transcriptomal changes induced by exposure to BPA and EE2, the similarity being more complete in the epithelial compartment. Because of these similarities in the transcriptomal responses of the developing mammary gland resulting from exposure to EE2 and BPA, we conclude that BPA acts as an estrogenic agent in this tissue. Recent publications have demonstrated estrogenic action of BPA in other tissues. For example, low-dose effects of BPA on insulin secretion are obliterated in the ERα and ERβ null mutant mice [36].

The mammary gland mesenchyme plays an inductive role on mammary epithelial morphogenesis [37]. From the present study, we conclude that the expression of ERα mRNA is detected only in the stromal compartment. The expression of both ERα and ERβ in the developing mammary glands has been well documented. Previous studies have shown that the expression of mRNAs for ERα and ERβ are first observed in the mesenchyme surrounding the mammary bud at E12.5 [25]. At E18, ERα is detected predominantly in the mammary mesenchyme and very few cells in the epithelium are ERα positive when examined by immunohistochemistry [13]. The expression of ERα shifts mainly to the epithelium only at post-natal time-points [38]. Although in the present study ERβ expression was not detected in the transcriptome of the stroma immediately adjacent to the epithelium (peri-ductal stroma), its involvement in BPA action cannot be ruled out, given that it was detected in RNA extracted from the whole fetal mammary gland at E18 [13] as well as from RNA extracted from the entire stroma and not just the peri-ductal region, at E19 (unpublished data). In the present study, the presence of the mRNA transcripts for *GPR30* in the peri-ductal stroma raises the possibility that BPA or EE2 could also exert their estrogenic effects through this receptor in addition to the classical nuclear ERs. However, this membrane receptor is only expressed at this developmental stage in the ERα(+/+) mice, thus, the estrogenic effects observed in the ERα(−/−) mice cannot be attributed to GPR30 action. The expression of the estrogen receptors in the stroma along with undetectable levels of these receptors in the epithelium makes the stroma a likely estrogen target at this period of mammary development. We further propose that the stroma may integrate the estrogenic input into a common set of mediators of stromal-epithelial interactions that in turn influence the epithelium.

While gene clusters 1 and 4 of the hierarchical cluster analysis show very similar expression patterns in BPA and EE2 exposed mice, clusters 2, 3, and 5 demonstrate differential effects of the two estrogens. This may be due to actions of BPA on nuclear receptors other than ERα and β that bind BPA and not EE2. These include Nr3c1 [39], Ar [40] and Thra [41]. Expression of each of these receptors has been confirmed in the fetal mammary gland in the present study. It is however important to note that binding affinity of BPA to Ar [42] and Thra [43] is 10-fold and 100-fold lower respectively relative to the classical ERs.

In the peri-ductal stroma, there was a decrease in expression of the mRNA transcripts for *Tnc* and *Vcan,* which are known to modulate the biomechanical properties of the extracellular matrix [44], [45] as well as cell adhesion [46]. Similarly, increased expression of genes involved in adipogenesis such as *Fabp4*, *PPARγ* and *Ldlr* was also consistent with our previous study showing increased adipogenesis in the periductal stroma of BPA-exposed fetal mammary glands [13]. Accelerated adipogenesis may be the underlying cause of increased epithelial area and branching in these fetal glands [24]. This is consistent with the well known property of the preadipocytes to promote epithelial elongation and branching [47]. In the epithelium, the increased expression of anti-apoptotic genes in the BPA-exposed mice was also consistent with our earlier observations showing inhibition of lumen formation [13]. Apoptosis in the ductal cords plays a critical role in lumen formation during mammary development [37]. These changes in gene expression patterns in the peri-ductal stroma and epithelium brought about by BPA exposure correlate with the subtle morphological changes previously observed in our lab. More importantly, they suggest altered stromal-epithelial interactions in the fetal glands. These alterations during fetal development may contribute to the prominent changes in the morphology of the mammary glands observed during adulthood, including increased ductal density and branching [10], [48]. In addition, the altered expression of genes involved in the focal adhesion pathway indicates potential changes in biomechanical properties of the peri-ductal stroma.

Whereas targeted deficiency of ERα causes strong phenotypic changes in the mouse mammary gland during its peripubertal development [49], no significant global transcriptomal differences were observed between the ERα(+/+) and the ERα(−/−) animals treated with vehicle or either estrogen in either compartment of the mammary glands at E19. It may hence be likely that these hormone mediated changes may occur through ERβ. However, by comparing the effect of the two estrogens in ERα(+/+) and the ERα(−/−) animals, we were able to identify putative ERα-dependent and estrogen responsive genes that are relevant to mammary gland development. These presumed ERα-dependent effects of BPA on the expression of several genes involved in mouse mammary gland development also point to altered stromal-epithelial interactions. In our microarray data, the expression of *Met* was up-regulated in the epithelium of mice exposed to BPA or EE2. *Met* encodes the membrane receptor of HGF, which is secreted in the stroma; ligand activation of the MET protein increases ductal branching [28]. This is consistent with our previous observation that ductal development and branching in the fetal mammary gland was enhanced in BPA-exposed mice [13]. In addition, increased expression of *Tgm2*, known to catalyze cross-linking of collagen-I [29], [30] is consistent with our previous observations of increased density of collagen fibers in the periductal stroma of BPA-exposed fetal mouse mammary glands [13]. In turn, this may be responsible for an increased extracellular matrix rigidity, which has been shown to inhibit lumen formation in other models [50], [51]. Thus, it is likely that *Tgm2* may indirectly mediate the inhibition of lumen formation which has been observed in the epithelium of BPA-exposed fetal mice [13].

In summary, perinatal exposure to BPA alters the stromal-epithelial interactions in the fetal mouse mammary gland. Stromal cells might respond to various estrogenic compounds with distinct transcriptomal changes and integrate such stimuli into a common (but yet to be identified) mediator(s) of mammary gland ductal morphogenesis. Possible involvement of estrogen receptors other than ERα on these BPA effects cannot be ruled out. However, the dependence of GPR30 on ERα expression suggests that GPR30 may not mediate the global transcriptomal changes observed herein. On the other hand, ERα-mediated BPA action may also alter the expression of a few key genes that regulate mammary gland development. Our results lend further support to the vulnerability of the fetus to xenoestrogen exposure that results in abnormalities that manifest during adult life [19], [52].

## Supporting Information

Figure S1 **Ductal outgrowth in mammary glands of CD1 and C57BL/6 mice.** A comparison of ductal growth in whole mounts of fetal mammary glands of CD1 and C57BL/6 mice at E16 through E19. Arrows indicate mammary buds on the skin. *Primary ductal outgrowth observed. Scale bar = 400 µm.
(TIF)

Figure S2 **Morphology of the fetal mammary gland at E19.** Whole mount (A) and stained section (B) of the fourth inguinal mammary gland of a female C57Bl/6 mouse at E19. The epithelial ducts and the peri-ductal stroma within 100 µm of the epithelium, outlined in yellow, were collected separately by laser capture microdissection (LN: lymph node; FP: presumptive fat pad; E: epithelium; Scale bar: 500 µm).
(JPG)

Figure S3 **Expression of ERα in the peri-ductal stroma.** Graph showing the expression of ERα (Esr1) in the peri-ductal stroma of ERα(+/+) and ERα(−/−) mice. (Arbitrary units: signal intensity observed in the microarray).
(TIF)

Table S1 **Morphometric properties of CD1 and C57BL/6 fetal mammary glands.**
(DOC)

Table S2 **Differentially regulated transcripts: Hierarchical Clustering Analysis.**
(DOC)

Table S3 **Transcripts regulated by ERα.**
(DOC)
