# Supplementary figures and images for: Low-Dose BPA Exposure Alters the Mesenchymal and Epithelial Transcriptomes of the Mouse Fetal Mammary Gland
Source: PLoS One. 2013 May 21;8(5):e63902. doi: 10.1371/journal.pone.0063902 (PMC3660582; doi:10.1371/journal.pone.0063902)

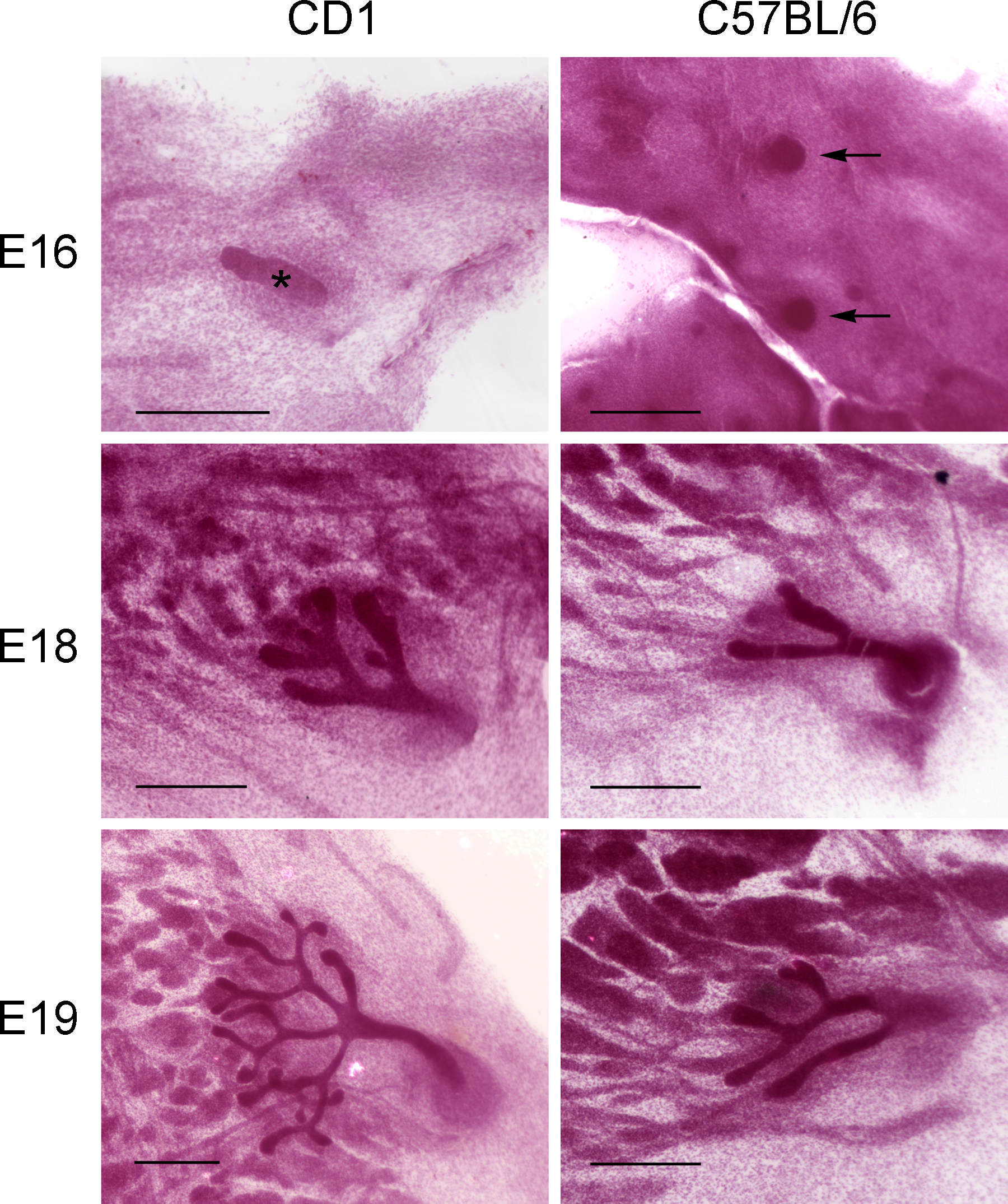

Supplement: Figure S1 — Ductal outgrowth in mammary glands of CD1 and C57BL/6 mice. A comparison of ductal growth in whole mounts of fetal mammary glands of CD1 and C57BL/6 mice at E16 through E19. Arrows indicate mammary buds on the skin. *Primary ductal outgrowth observed. Scale bar = 400 µm. (TIF) [file pone.0063902.s001.tif]

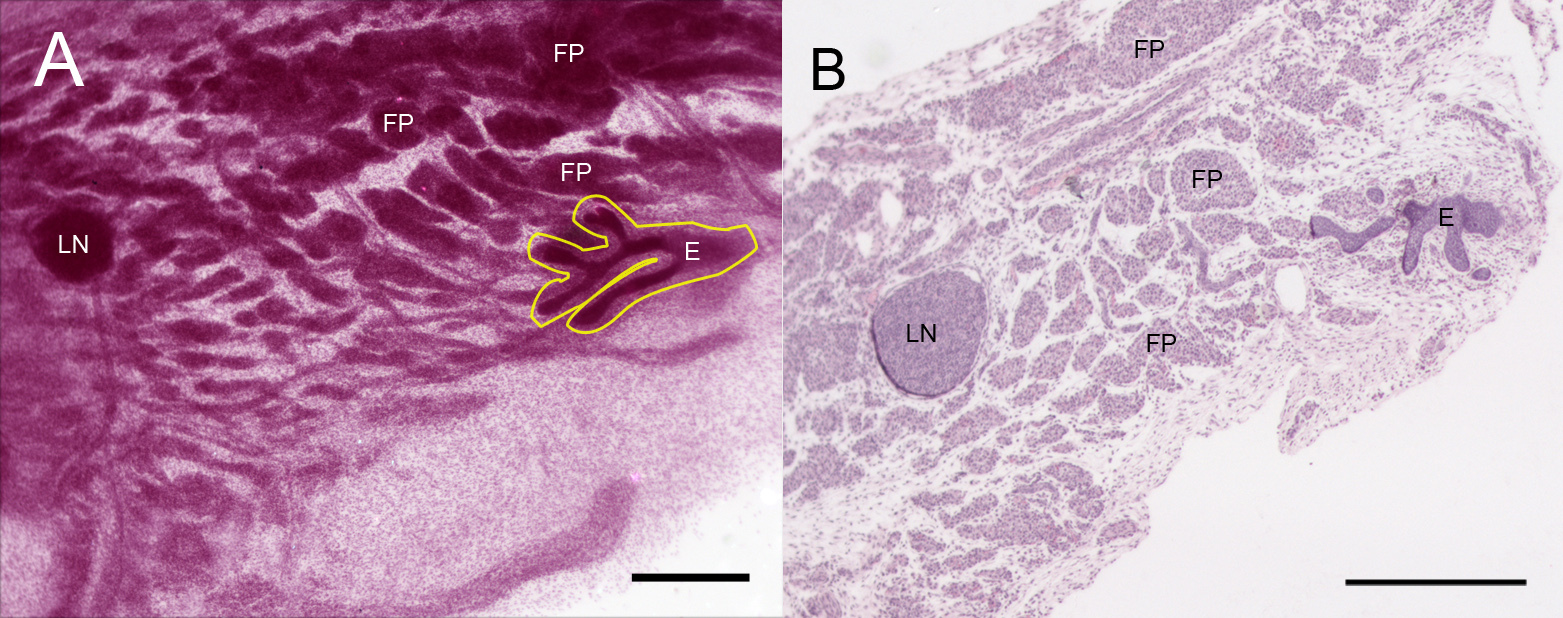

Supplement: Figure S2 — Morphology of the fetal mammary gland at E19. Whole mount (A) and stained section (B) of the fourth inguinal mammary gland of a female C57Bl/6 mouse at E19. The epithelial ducts and the peri-ductal stroma within 100 µm of the epithelium, outlined in yellow, were collected separately by laser capture microdissection (LN: lymph node; FP: presumptive fat pad; E: epithelium; Scale bar: 500 µm). (JPG) [file pone.0063902.s002.jpg]

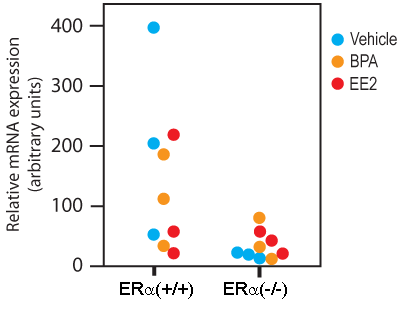

Supplement: Figure S3 — Expression of ERα in the peri-ductal stroma. Graph showing the expression of ERα (Esr1) in the peri-ductal stroma of ERα(+/+) and ERα(−/−) mice. (Arbitrary units: signal intensity observed in the microarray). (TIF) [file pone.0063902.s003.tif]
