# Supplementary material for: Low-Dose BPA Exposure Alters the Mesenchymal and Epithelial Transcriptomes of the Mouse Fetal Mammary Gland
Source: PLoS One. 2013 May 21;8(5):e63902. doi: 10.1371/journal.pone.0063902 (PMC3660582; doi:10.1371/journal.pone.0063902)
Supplement: Table S1 — Morphometric properties of CD1 and C57BL/6 fetal mammary glands. (DOC) [file pone.0063902.s004.doc]

**Table S1: Morphometric properties of CD1 and C57BL/6 fetal mammary glands**

|  | **E18 CD1** | **E18 BL/6** | **E19 CD1** | **E19 BL/6** |
| --- | --- | --- | --- | --- |
| **Area subtended by ducts (µm^2^)** | 13964.78+5455.33 ^a^ | 7553.74+3489.25 ^b^ | 34768.22+12106.13 ^c^ | 14444.69+3831.25 ^a^ |
| **Ductal area (µm^2^)** | 7351.67+2187.18 ^a^ | 4576.18+1668.31 ^b^ | 13689.25+3348.63 ^c^ | 7273.05+1787.89 ^a^ |
| **Ductal length (µm)** | 183.21+43.18 ^a^ | 144.67+47.70 ^b^ | 260.51+59.04 ^c^ | 158.07+65.71 ^a b^ |
| **# branching points** | 3.40+1.47 ^a^ | 2.29+1.38 ^a^ | 7.45+2.77 ^b^ | 3.33+1.05 ^a^ |
| **# terminal ends** | 4.44+1.56 ^a^ | 3.29+1.38 ^a^ | 8.91+2.98 ^b^ | 4.47+0.99 ^a^ |

A comparison of the morphometric properties of ductal structures in the mammary glands of CD1 and C57BL/6 mice at E16, E18 and E19. (Differing alphabets indicate significant differences analyzed by the t-test, p<0.05)
