# Supplementary material for: Low-Dose BPA Exposure Alters the Mesenchymal and Epithelial Transcriptomes of the Mouse Fetal Mammary Gland
Source: PLoS One. 2013 May 21;8(5):e63902. doi: 10.1371/journal.pone.0063902 (PMC3660582; doi:10.1371/journal.pone.0063902)
Supplement: Table S2 — Differentially regulated transcripts: Hierarchical Clustering Analysis. (DOC) [file pone.0063902.s005.doc]

**Table S2: Differentially regulated transcripts: Hierarchical Clustering Analysis**

| Cluster # | Expression in exposure group | | | Number of Genes in Stroma | Number of Genes in Epithelium |
| --- | --- | --- | --- | --- | --- |
|  | Vehicle | BPA | EE2 |  |  |
| 1 | + | - | - | 790 | 1218 |
| 2 | + | + | - | 165 | 136 |
| 3 | - | - | + | 243 | 134 |
| 4 | - | + | + | 457 | 3671 |
| 5 | - | + | - | 381 | 189 |

The number of differentially regulated transcripts in the gene clusters resulting from hierarchical clustering analysis. [(+) Up or (-) down-regulated genes compared to vehicle control]
