# Supplementary material for: Low-Dose BPA Exposure Alters the Mesenchymal and Epithelial Transcriptomes of the Mouse Fetal Mammary Gland
Source: PLoS One. 2013 May 21;8(5):e63902. doi: 10.1371/journal.pone.0063902 (PMC3660582; doi:10.1371/journal.pone.0063902)
Supplement: Table S3 — Transcripts regulated by ERα. (DOC) [file pone.0063902.s006.doc]

**Table S3:** **Transcripts regulated by ERα**

**Table S3a:** **Transcripts regulated by ERα that are down-regulated in the epithelium**

|  | **ERα(+/+)** | | | **ERα(-/-)** | | |  |
| --- | --- | --- | --- | --- | --- | --- | --- |
| **Gene Symbol** | **Veh/BPA** | **Veh/EE2** | **highest ratio** | **Veh/BPA** | **Veh/EE2** | **highest ratio** |  |
| Mbd1 | 3.1 | 5.4 | 5.4 | 0.6 | 0.9 | 0.9 |  |
| B430203M17Rik | 1.1 | 4.2 | 4.2 | 0.4 | 0.6 | 0.6 |  |
| Zfp90 | 1.4 | 4.0 | 4.0 | 1.0 | 0.8 | 1.0 |  |
| Tmem65 | 2.1 | 2.9 | 2.9 | 0.5 | 0.4 | 0.5 |  |
| Stag1 | 1.9 | 2.9 | 2.9 | 0.6 | 0.6 | 0.6 |  |
| 1810030O07Rik | 1.1 | 2.6 | 2.6 | 0.4 | 0.4 | 0.4 |  |
| Dbndd2 | 2.0 | 2.8 | 2.8 | 0.9 | 0.8 | 0.9 |  |
| Fem1a | 1.1 | 2.6 | 2.6 | 0.6 | 0.8 | 0.8 |  |
| Zyx | 1.5 | 2.6 | 2.6 | 0.7 | 0.8 | 0.8 |  |
| Rraga | 1.6 | 2.3 | 2.3 | 0.6 | 0.5 | 0.6 |  |
| Ccdc109a | 1.2 | 2.2 | 2.2 | 0.4 | 0.5 | 0.5 |  |
| Prosc | 1.1 | 2.3 | 2.3 | 0.6 | 0.6 | 0.6 |  |
| Gemin5 | 0.9 | 2.3 | 2.3 | 0.9 | 0.8 | 0.9 |  |
| Zcchc9 | 1.8 | 2.6 | 2.6 | 1.1 | 1.1 | 1.1 |  |
| Mapk6 | 0.7 | 2.0 | 2.0 | 0.4 | 0.5 | 0.5 |  |
| Cpsf1 | 1.1 | 2.4 | 2.4 | 0.9 | 0.6 | 0.9 |  |
| Ciapin1 | 1.1 | 2.1 | 2.1 | 0.6 | 0.5 | 0.6 |  |
| BC037112 | 1.5 | 2.0 | 2.0 | 0.6 | 0.5 | 0.6 |  |
| Peli1 | 1.5 | 2.0 | 2.0 | 0.6 | 0.5 | 0.6 |  |
| Rnf7 | 2.1 | 1.8 | 2.1 | 0.7 | 0.6 | 0.7 |  |
| Phactr4 | 1.1 | 2.2 | 2.2 | 0.8 | 0.6 | 0.8 |  |
| Tpd52 | 1.3 | 2.5 | 2.5 | 1.0 | 1.2 | 1.2 |  |
| Zmynd11 | 0.9 | 2.0 | 2.0 | 0.7 | 0.8 | 0.8 |  |
| Oraov1 | 1.0 | 2.0 | 2.0 | 0.7 | 0.8 | 0.8 |  |
| Npepps | 1.2 | 2.0 | 2.0 | 0.8 | 0.6 | 0.8 |  |
| Dera | 0.6 | 2.0 | 2.0 | 0.8 | 0.7 | 0.8 |  |
| Farp1 | 1.1 | 2.0 | 2.0 | 1.0 | 0.8 | 1.0 |  |
| 1110039B18Rik | 1.3 | 2.3 | 2.3 | 1.1 | 1.4 | 1.4 |  |
| Josd3 | 1.5 | 2.0 | 2.0 | 1.1 | 0.9 | 1.1 |  |
| Huwe1 | 1.1 | 2.2 | 2.2 | 1.2 | 1.3 | 1.3 |  |
| Gja1 | 1.7 | 2.0 | 2.0 | 0.8 | 1.1 | 1.1 |  |
| BC038286 | 1.3 | 2.0 | 2.0 | 1.1 | 1.2 | 1.2 |  |

Genes with only 2-fold decrease in expression, between vehicle and estrogen treatments are listed.

Transcripts regulated by ERα (genes down-regulated in the epithelium of ERα(+/+) but not in ERα(-/-) mice).

**Table S3b:** **Transcripts regulated by ERα that are up-regulated in the epithelium**

|  | **ERα(+/+)** | | | **ERα(-/-)** | | |  |
| --- | --- | --- | --- | --- | --- | --- | --- |
| **Gene Symbol** | **BPA/Veh** | **EE2/Veh** | **highest ratio** | **BPA/Veh** | **EE2/Veh** | **highest ratio** |  |
| Col2a1 | 1.8 | 5.5 | 5.5 | 0.9 | 1.1 | 1.1 |  |
| Zbtb7a | 5.4 | 5.6 | 5.6 | 1.3 | 0.8 | 1.3 |  |
| Tpx2 | 5.0 | 1.4 | 5.0 | 1.1 | 1.1 | 1.1 |  |
| Dcpp1 /// Dcpp2 /// Dcpp3 /// LOC100046800 | 3.4 | 1.4 | 3.4 | 0.7 | 0.9 | 0.9 |  |
| 1700123O20Rik | 3.2 | 1.5 | 3.2 | 0.8 | 0.9 | 0.9 |  |
| Tgm2 | 2.4 | 3.0 | 3.0 | 0.7 | 0.7 | 0.7 |  |
| Cdk9 | 2.4 | 0.9 | 2.4 | 0.3 | 0.3 | 0.3 |  |
| Met | 1.7 | 2.9 | 2.9 | 1.1 | 0.9 | 1.1 |  |
| Sbf2 | 2.3 | 2.9 | 2.9 | 1.0 | 1.1 | 1.1 |  |
| Nr2f6 | 2.5 | 0.7 | 2.5 | 0.7 | 0.6 | 0.7 |  |
| 0610006I08Rik | 2.3 | 0.9 | 2.3 | 0.5 | 0.4 | 0.5 |  |
| 1110005A23Rik /// EG625193 | 2.1 | 3.2 | 3.2 | 1.5 | 1.0 | 1.5 |  |
| Ergic3 | 2.7 | 2.4 | 2.7 | 1.0 | 0.9 | 1.0 |  |
| Gtf2h4 | 1.2 | 2.6 | 2.6 | 0.9 | 0.7 | 0.9 |  |
| B230354K17Rik | 2.8 | 1.9 | 2.8 | 1.1 | 1.2 | 1.2 |  |
| LOC100044468 /// Nlk | 2.8 | 1.1 | 2.8 | 1.2 | 1.1 | 1.2 |  |
| Atp6v1a | 2.3 | 2.6 | 2.6 | 1.0 | 1.0 | 1.0 |  |
| Pcm1 | 1.3 | 2.6 | 2.6 | 1.1 | 1.1 | 1.1 |  |
| Exod1 | 2.3 | 1.2 | 2.3 | 0.9 | 0.6 | 0.9 |  |
| Sulf2 | 2.2 | 1.2 | 2.2 | 0.8 | 0.6 | 0.8 |  |
| 4921506J03Rik | 2.6 | 1.5 | 2.6 | 1.2 | 1.2 | 1.2 |  |
| C80913 | 2.2 | 1.0 | 2.2 | 0.8 | 0.8 | 0.8 |  |
| Exosc6 | 1.3 | 2.4 | 2.4 | 0.9 | 1.0 | 1.0 |  |
| Cebpb | 3.2 | 1.8 | 3.2 | 1.8 | 1.1 | 1.8 |  |
| Ergic3 | 2.2 | 2.0 | 2.2 | 0.8 | 0.8 | 0.8 |  |
| Apod | 2.5 | 1.7 | 2.5 | 0.8 | 1.1 | 1.1 |  |
| Magoh | 1.4 | 2.1 | 2.1 | 0.8 | 0.6 | 0.8 |  |
| Pold1 | 1.3 | 2.3 | 2.3 | 0.8 | 1.0 | 1.0 |  |
| Cdt1 | 2.5 | 1.3 | 2.5 | 1.2 | 1.2 | 1.2 |  |
| Snapc2 | 1.6 | 2.1 | 2.1 | 0.8 | 0.7 | 0.8 |  |
| Hnrnpl | 2.7 | 1.5 | 2.7 | 1.5 | 1.4 | 1.5 |  |
| Ptar1 | 2.6 | 2.2 | 2.6 | 0.9 | 1.3 | 1.3 |  |
| Sema4c | 2.2 | 3.1 | 3.1 | 2.0 | 1.7 | 2.0 |  |
| Fbxw11 | 2.3 | 2.1 | 2.3 | 1.2 | 1.1 | 1.2 |  |
| Fbl /// LOC100044829 | 2.6 | 1.3 | 2.6 | 1.3 | 1.4 | 1.4 |  |
| Egln3 | 2.2 | 1.2 | 2.2 | 0.9 | 1.1 | 1.1 |  |
| Thrap3 | 2.4 | 1.3 | 2.4 | 1.3 | 1.3 | 1.3 |  |
| D17H6S56E-5 | 2.3 | 2.1 | 2.3 | 1.2 | 1.2 | 1.2 |  |
| Pcm1 | 1.6 | 2.4 | 2.4 | 1.2 | 1.3 | 1.3 |  |
| Trim27 | 2.1 | 1.8 | 2.1 | 0.9 | 1.0 | 1.0 |  |
| Ascc3l1 | 1.7 | 2.4 | 2.4 | 1.4 | 1.4 | 1.4 |  |
| 1810035L17Rik | 2.0 | 1.1 | 2.0 | 1.0 | 0.9 | 1.0 |  |
| Ngdn | 1.8 | 2.0 | 2.0 | 1.0 | 0.5 | 1.0 |  |
| 2310051E17Rik /// Klf9 | 1.6 | 2.2 | 2.2 | 1.2 | 1.0 | 1.2 |  |
| Hnrpab | 2.9 | 1.3 | 2.9 | 1.0 | 1.9 | 1.9 |  |
| Aes | 1.0 | 2.0 | 2.0 | 1.0 | 1.0 | 1.0 |  |
| Ldhb | 2.1 | 1.6 | 2.1 | 1.1 | 1.0 | 1.1 |  |
| C1qdc2 | 2.1 | 1.0 | 2.1 | 1.1 | 1.0 | 1.1 |  |
| Eif4a1 | 2.1 | 1.3 | 2.1 | 1.0 | 1.1 | 1.1 |  |
| Tcf7l2 | 2.0 | 1.3 | 2.0 | 1.0 | 1.1 | 1.1 |  |
| 2410015M20Rik | 2.8 | 1.9 | 2.8 | 1.9 | 1.4 | 1.9 |  |
| Zfp313 | 2.2 | 2.1 | 2.2 | 1.3 | 1.3 | 1.3 |  |
| Taf10 | 1.2 | 2.0 | 2.0 | 1.1 | 1.2 | 1.2 |  |
| Ltbp3 | 2.3 | 1.2 | 2.3 | 1.5 | 1.3 | 1.5 |  |
| Ash1l | 1.3 | 2.1 | 2.1 | 1.2 | 1.3 | 1.3 |  |
| 3110003A17Rik | 2.0 | 2.0 | 2.0 | 1.2 | 1.0 | 1.2 |  |
| LOC100048499 /// Mfap1a /// Mfap1b | 2.2 | 1.2 | 2.2 | 1.4 | 1.3 | 1.4 |  |
| Nfic | 2.3 | 1.7 | 2.3 | 1.5 | 0.9 | 1.5 |  |
| Mrpl41 | 2.0 | 1.9 | 2.0 | 1.3 | 0.9 | 1.3 |  |
| Sucla2 | 2.9 | 2.6 | 2.9 | 2.1 | 1.5 | 2.1 |  |
| Hnrpul1 | 2.0 | 1.7 | 2.0 | 1.2 | 1.2 | 1.2 |  |
| Cnih | 1.4 | 2.1 | 2.1 | 1.3 | 1.0 | 1.3 |  |
| Tnk2 | 2.0 | 0.8 | 2.0 | 1.2 | 1.1 | 1.2 |  |
| Rbm5 | 1.2 | 2.4 | 2.4 | 1.7 | 1.6 | 1.7 |  |
| Hectd1 | 2.0 | 1.2 | 2.0 | 1.3 | 1.2 | 1.3 |  |
| 2810482I07Rik | 2.1 | 1.6 | 2.1 | 1.4 | 1.3 | 1.4 |  |
| D3Ertd300e | 2.3 | 1.1 | 2.3 | 1.6 | 1.6 | 1.6 |  |
| Edf1 /// LOC674653 | 1.6 | 2.1 | 2.1 | 1.4 | 1.1 | 1.4 |  |
| Atp5a1 | 2.3 | 1.1 | 2.3 | 1.6 | 1.1 | 1.6 |  |
| Pttg1ip | 2.2 | 0.8 | 2.2 | 1.5 | 1.2 | 1.5 |  |
| Aqp5 /// LOC100046616 | 2.0 | 1.0 | 2.0 | 1.3 | 1.3 | 1.3 |  |
| LOC100043295 /// LOC100047935 /// LOC382740 /// LOC632230 /// LOC664956 /// LOC666545 /// LOC675821 /// Rpl5 | 1.5 | 2.0 | 2.0 | 1.3 | 1.3 | 1.3 |  |
| Depdc6 | 2.1 | 1.9 | 2.1 | 1.3 | 1.5 | 1.5 |  |
| Derl2 | 2.2 | 1.3 | 2.2 | 1.5 | 1.4 | 1.5 |  |
| Cnot4 | 2.4 | 1.9 | 2.4 | 1.3 | 1.7 | 1.7 |  |
| Arhgdib | 1.2 | 2.3 | 2.3 | 1.3 | 1.7 | 1.7 |  |
| Nedd8 | 2.3 | 2.0 | 2.3 | 1.7 | 1.5 | 1.7 |  |
| LOC100042343 /// Tmed2 | 2.0 | 1.7 | 2.0 | 1.3 | 1.2 | 1.3 |  |
| Nfix | 2.0 | 1.2 | 2.0 | 1.3 | 1.3 | 1.3 |  |
| BC005512 /// EG641366 /// LOC215866 /// LOC629242 | 1.7 | 2.0 | 2.0 | 1.4 | 1.3 | 1.4 |  |
| D17H6S56E-5 | 2.2 | 1.5 | 2.2 | 1.6 | 1.5 | 1.6 |  |
| Gtpbp4 | 2.4 | 1.2 | 2.4 | 1.8 | 1.5 | 1.8 |  |
| LOC100046746 /// Smap1 | 2.2 | 1.3 | 2.2 | 1.6 | 1.4 | 1.6 |  |
| Hnrnpa1 | 2.2 | 1.5 | 2.2 | 1.7 | 1.6 | 1.7 |  |
| A430105D02Rik | 3.0 | 2.4 | 3.0 | 2.2 | 2.5 | 2.5 |  |
| Col4a2 | 2.2 | 1.7 | 2.2 | 1.7 | 1.5 | 1.7 |  |
| Cwc15 | 2.1 | 1.7 | 2.1 | 1.6 | 1.3 | 1.6 |  |
| Samm50 | 2.0 | 1.3 | 2.0 | 1.5 | 1.3 | 1.5 |  |
| Akr1a4 | 2.0 | 1.5 | 2.0 | 1.6 | 1.6 | 1.6 |  |
| Arl4c /// LOC632433 | 2.0 | 1.2 | 2.0 | 1.5 | 1.2 | 1.5 |  |
| Tlk2 | 2.0 | 1.2 | 2.0 | 1.5 | 1.6 | 1.6 |  |
| Anapc5 | 2.5 | 1.8 | 2.5 | 2.1 | 2.0 | 2.1 |  |
| Ehmt2 | 2.0 | 2.0 | 2.0 | 1.6 | 1.5 | 1.6 |  |
| Cldn12 | 2.0 | 1.4 | 2.0 | 1.6 | 1.2 | 1.6 |  |
| Ttc14 | 2.5 | 1.4 | 2.5 | 1.8 | 2.1 | 2.1 |  |
| Angel2 | 2.2 | 1.2 | 2.2 | 1.6 | 1.8 | 1.8 |  |
| Rbm5 | 1.5 | 2.2 | 2.2 | 1.9 | 1.8 | 1.9 |  |
| 1110034B05Rik | 1.6 | 3.2 | 3.2 | 2.9 | 1.8 | 2.9 |  |
| Anapc5 | 2.3 | 1.9 | 2.3 | 2.1 | 2.0 | 2.1 |  |
| Jmy | 2.0 | 1.4 | 2.0 | 1.5 | 1.8 | 1.8 |  |
| 2410003K15Rik | 2.1 | 1.2 | 2.1 | 2.0 | 1.2 | 2.0 |  |
| Mrps15 | 2.1 | 1.2 | 2.1 | 1.9 | 2.0 | 2.0 |  |
| Ndrg2 | 2.0 | 1.8 | 2.0 | 1.9 | 1.7 | 1.9 |  |
| Rab6 | 1.5 | 2.1 | 2.1 | 2.0 | 1.8 | 2.0 |  |
| Got2 | 2.4 | 1.2 | 2.4 | 2.0 | 2.3 | 2.3 |  |
| Rrp1 | 2.1 | 1.5 | 2.1 | 2.0 | 2.1 | 2.1 |  |
| Arhgef12 | 2.0 | 1.9 | 2.0 | 1.9 | 1.7 | 1.9 |  |
| Ssrp1 | 1.7 | 2.1 | 2.1 | 1.8 | 2.0 | 2.0 |  |
| Ldhb | 2.0 | 1.5 | 2.0 | 2.0 | 1.7 | 2.0 |  |
| 4933439C20Rik | 2.0 | 1.4 | 2.0 | 2.0 | 1.8 | 2.0 |  |
| Arf3 | 2.0 | 1.6 | 2.0 | 1.8 | 2.0 | 2.0 |  |
| Phlda1 | 2.7 | 1.4 | 2.7 | 2.3 | 2.7 | 2.7 |  |
| Pcbd2 | 2.2 | 2.3 | 2.3 | 2.3 | 2.1 | 2.3 |  |
| Ccdc55 | 1.9 | 2.0 | 2.0 | 1.8 | 2.1 | 2.1 |  |
| Urod | 1.2 | 2.3 | 2.3 | 2.4 | 1.9 | 2.4 |  |
| Uqcrc1 | 2.0 | 1.9 | 2.0 | 2.1 | 1.6 | 2.1 |  |
| Ndufb9 | 2.2 | 1.3 | 2.2 | 2.3 | 1.9 | 2.3 |  |
| 2810004N23Rik /// Setd6 | 1.6 | 2.0 | 2.0 | 1.7 | 2.1 | 2.1 |  |
| Ik | 2.4 | 1.8 | 2.4 | 2.6 | 2.0 | 2.6 |  |
| Synpo | 1.8 | 2.1 | 2.1 | 2.2 | 2.2 | 2.2 |  |
| Ssrp1 | 1.5 | 2.0 | 2.0 | 2.0 | 2.2 | 2.2 |  |
| Ipo7 | 2.0 | 1.3 | 2.0 | 1.4 | 2.4 | 2.4 |  |
| Gtf2f1 | 2.1 | 1.4 | 2.1 | 2.0 | 2.5 | 2.5 |  |
| Ctcf | 2.0 | 1.5 | 2.0 | 2.4 | 1.9 | 2.4 |  |
| 2310067E19Rik | 1.7 | 2.0 | 2.0 | 2.5 | 2.5 | 2.5 |  |
| Krr1 | 1.5 | 2.3 | 2.3 | 2.6 | 2.8 | 2.8 |  |
| Atp5b | 2.2 | 1.7 | 2.2 | 2.6 | 2.2 | 2.6 |  |
| Ghitm | 2.4 | 1.0 | 2.4 | 2.9 | 2.3 | 2.9 |  |
| Sf3b2 | 2.0 | 1.2 | 2.0 | 2.5 | 2.5 | 2.5 |  |
| Sfrs9 | 1.8 | 2.0 | 2.0 | 2.0 | 2.6 | 2.6 |  |
| Grn | 2.0 | 1.7 | 2.0 | 1.7 | 2.6 | 2.6 |  |
| Fus | 2.1 | 1.9 | 2.1 | 2.7 | 2.3 | 2.7 |  |
| Uqcrh | 2.6 | 1.9 | 2.6 | 3.4 | 2.7 | 3.4 |  |
| Hadhb | 2.2 | 0.8 | 2.2 | 3.1 | 1.6 | 3.1 |  |
| Nfix | 2.6 | 1.8 | 2.6 | 3.0 | 3.4 | 3.4 |  |
| Uqcrc2 | 2.1 | 1.7 | 2.1 | 3.0 | 2.0 | 3.0 |  |
| Aco2 | 2.0 | 0.8 | 2.0 | 2.9 | 1.5 | 2.9 |  |
| Ppp1r3b | 2.1 | 1.1 | 2.1 | 3.0 | 2.5 | 3.0 |  |
| Arl4a | 2.2 | 1.3 | 2.2 | 3.2 | 2.0 | 3.2 |  |
| Fh1 | 2.0 | 1.7 | 2.0 | 3.2 | 2.2 | 3.2 |  |
| Slc9a3r2 | 2.8 | 2.0 | 2.8 | 3.2 | 4.0 | 4.0 |  |
| Slc9a3r2 | 2.7 | 2.2 | 2.7 | 3.0 | 3.9 | 3.9 |  |
| 4632417K18Rik | 1.2 | 2.3 | 2.3 | 3.6 | 3.5 | 3.6 |  |
| Rbm5 | 1.7 | 2.0 | 2.0 | 3.5 | 2.8 | 3.5 |  |
| Zc3h13 | 3.0 | 1.2 | 3.0 | 4.6 | 4.3 | 4.6 |  |
| Ets2 | 2.0 | 1.6 | 2.0 | 3.7 | 3.0 | 3.7 |  |
| Adamts4 | 2.8 | 2.3 | 2.8 | 2.7 | 4.5 | 4.5 |  |
| Cebpa | 2.1 | 1.0 | 2.1 | 4.0 | 2.6 | 4.0 |  |

Genes with only 2-fold increase in expression, between vehicle and estrogen treatments are listed.

Transcripts regulated by ERα (genes up-regulated in the epithelium of ERα(+/+) but not in ERα(-/-) mice).

**Table S3c:** **Transcripts regulated by ERα that are down-regulated in the peri-ductal stroma**

|  | **ERα(+/+)** | | | **ERα(-/-)** | | |  |
| --- | --- | --- | --- | --- | --- | --- | --- |
| **Gene Symbol** | **Veh/BPA** | **Veh/EE2** | **highest ratio** | **Veh/BPA** | **Veh/EE2** | **highest ratio** |  |
| Trim29 | 4.0 | 4.0 | 4.0 | 0.6 | 0.9 | 0.9 |  |
| Spna2 | 1.6 | 3.3 | 3.3 | 0.8 | 1.0 | 1.0 |  |
| Sc4mol | 1.3 | 2.9 | 2.9 | 0.6 | 1.0 | 1.0 |  |
| Cnn3 /// LOC100047856 | 3.0 | 1.9 | 3.0 | 1.0 | 1.3 | 1.3 |  |
| ENSMUSG00000073645 | 2.2 | 1.7 | 2.2 | 0.3 | 0.6 | 0.6 |  |
| 1200016E24Rik | 2.6 | 1.0 | 2.6 | 0.8 | 1.0 | 1.0 |  |
| Casc4 | 1.5 | 2.6 | 2.6 | 0.9 | 1.1 | 1.1 |  |
| 4933413G19Rik /// Foxm1 | 1.7 | 2.9 | 2.9 | 1.1 | 1.4 | 1.4 |  |
| Gpr116 | 1.4 | 2.5 | 2.5 | 0.9 | 1.1 | 1.1 |  |
| LOC552906 | 2.1 | 3.3 | 3.3 | 1.6 | 2.0 | 2.0 |  |
| Ncapg | 1.2 | 2.2 | 2.2 | 0.6 | 0.9 | 0.9 |  |
| Fcer1g | 1.6 | 2.3 | 2.3 | 0.9 | 1.0 | 1.0 |  |
| Hmgcs1 /// LOC100040592 | 1.5 | 2.2 | 2.2 | 0.7 | 0.9 | 0.9 |  |
| 4933439C20Rik | 1.9 | 2.4 | 2.4 | 1.0 | 1.1 | 1.1 |  |
| Scarb1 | 0.7 | 2.1 | 2.1 | 0.7 | 0.8 | 0.8 |  |
| Cnn3 /// LOC100047856 | 2.5 | 1.7 | 2.5 | 1.0 | 1.2 | 1.2 |  |
| Appbp2 | 0.9 | 2.1 | 2.1 | 0.9 | 0.9 | 0.9 |  |
| Atpbd4 | 1.1 | 2.7 | 2.7 | 1.4 | 1.5 | 1.5 |  |
| Mxra8 | 2.4 | 1.0 | 2.4 | 1.2 | 1.3 | 1.3 |  |
| Ssbp3 | 2.0 | 1.2 | 2.0 | 0.4 | 0.9 | 0.9 |  |
| Atp7a | 2.0 | 1.3 | 2.0 | 0.8 | 0.9 | 0.9 |  |
| 4921506J03Rik | 1.2 | 2.0 | 2.0 | 0.9 | 0.9 | 0.9 |  |
| LOC100048397 | 2.4 | 1.7 | 2.4 | 0.8 | 1.4 | 1.4 |  |
| Pum2 | 1.5 | 2.3 | 2.3 | 0.8 | 1.3 | 1.3 |  |
| Txnl1 | 2.3 | 1.1 | 2.3 | 0.7 | 1.4 | 1.4 |  |
| Prpf6 | 2.3 | 2.1 | 2.3 | 1.3 | 1.4 | 1.4 |  |
| Thra | 1.3 | 2.0 | 2.0 | 1.1 | 1.1 | 1.1 |  |
| Gng12 | 2.0 | 1.9 | 2.0 | 0.9 | 1.1 | 1.1 |  |
| Lrrc17 | 2.4 | 1.2 | 2.4 | 1.2 | 1.5 | 1.5 |  |
| Raph1 | 2.1 | 1.6 | 2.1 | 0.8 | 1.2 | 1.2 |  |
| Atp6v0c /// Atp6v0c-ps2 | 1.3 | 2.0 | 2.0 | 1.1 | 1.1 | 1.1 |  |
| B230219D22Rik | 2.2 | 1.2 | 2.2 | 1.4 | 1.2 | 1.4 |  |
| Jak2 | 2.1 | 1.3 | 2.1 | 1.3 | 1.3 | 1.3 |  |
| LOC100039786 /// LOC676123 /// Ywhaq | 1.7 | 2.0 | 2.0 | 1.2 | 1.2 | 1.2 |  |
| Mrpl44 | 2.0 | 1.4 | 2.0 | 1.0 | 1.3 | 1.3 |  |
| Sfrs2 | 2.0 | 1.4 | 2.0 | 1.0 | 1.3 | 1.3 |  |
| Refbp2 /// Thoc4 | 2.4 | 1.9 | 2.4 | 0.8 | 1.8 | 1.8 |  |
| Fscn1 | 2.2 | 1.5 | 2.2 | 1.3 | 1.6 | 1.6 |  |
| Cald1 | 1.6 | 2.0 | 2.0 | 1.0 | 1.4 | 1.4 |  |
| Camk2n1 | 1.9 | 2.2 | 2.2 | 0.5 | 1.6 | 1.6 |  |
| Msn | 2.0 | 1.3 | 2.0 | 1.2 | 1.4 | 1.4 |  |
| Nhsl1 | 2.0 | 1.9 | 2.0 | 1.1 | 1.4 | 1.4 |  |
| Gas1 | 2.3 | 1.5 | 2.3 | 1.2 | 1.7 | 1.7 |  |
| Btg2 | 0.9 | 2.0 | 2.0 | 0.5 | 1.7 | 1.7 |  |
| Atrx | 2.2 | 1.5 | 2.2 | 1.6 | 2.2 | 2.2 |  |
| Trim27 | 0.8 | 2.0 | 2.0 | 1.2 | 2.0 | 2.0 |  |
| Igf2bp3 | 2.3 | 1.9 | 2.3 | 1.9 | 2.8 | 2.8 |  |

Genes with only 2-fold decrease in expression, between vehicle and estrogen treatments are listed.

Transcripts regulated by ERα (genes down-regulated in the peri-ductal stroma of ERα(+/+) but not in ERα(-/-) mice mice).

**Table S3d: Transcripts regulated by ERα that are up-regulated in the peri-ductal stroma**

|  | **ERα(+/+)** | | | **ERα(-/-)** | | |  |
| --- | --- | --- | --- | --- | --- | --- | --- |
| **Gene Symbol** | **BPA/Veh** | **EE2/Veh** | **highest ratio** | **BPA/Veh** | **EE2/Veh** | **highest ratio** |  |
| Gna12 | 5.4 | 1.6 | 5.4 | 1.2 | 0.5 | 1.2 |  |
| Lrrc41 | 2.5 | 2.8 | 2.8 | 0.6 | 0.5 | 0.6 |  |
| 3110037I16Rik | 2.3 | 3.0 | 3.0 | 1.2 | 1.3 | 1.3 |  |
| Dzip3 | 1.3 | 2.3 | 2.3 | 0.9 | 0.8 | 0.9 |  |
| Rnf215 | 2.7 | 0.6 | 2.7 | 1.3 | 0.4 | 1.3 |  |
| Cln8 | 2.1 | 1.4 | 2.1 | 0.6 | 0.7 | 0.7 |  |
| Spred1 | 0.8 | 2.3 | 2.3 | 1.0 | 0.9 | 1.0 |  |
| Cabin1 | 1.1 | 2.6 | 2.6 | 1.3 | 0.6 | 1.3 |  |
| Prpf31 | 2.6 | 2.7 | 2.7 | 1.4 | 1.5 | 1.5 |  |
| Depdc6 | 3.9 | 1.3 | 3.9 | 2.8 | 1.9 | 2.8 |  |
| Crk | 1.5 | 2.2 | 2.2 | 1.1 | 0.9 | 1.1 |  |
| Pin1 | 1.5 | 2.1 | 2.1 | 1.0 | 0.6 | 1.0 |  |
| Pdgfa | 1.6 | 2.2 | 2.2 | 1.1 | 0.9 | 1.1 |  |
| Tcn2 | 2.4 | 2.0 | 2.4 | 1.3 | 0.7 | 1.3 |  |
| Acsl3 | 1.1 | 2.6 | 2.6 | 1.1 | 1.6 | 1.6 |  |
| Cox8a | 1.3 | 2.2 | 2.2 | 1.2 | 1.0 | 1.2 |  |
| Nsmce2 | 1.1 | 2.4 | 2.4 | 1.2 | 1.5 | 1.5 |  |
| Ptprd | 1.0 | 2.1 | 2.1 | 1.2 | 0.7 | 1.2 |  |
| Atp5k | 2.2 | 1.6 | 2.2 | 1.3 | 1.0 | 1.3 |  |
| Rps7 | 2.1 | 1.2 | 2.1 | 1.2 | 1.2 | 1.2 |  |
| St7l | 2.5 | 0.6 | 2.5 | 1.6 | 1.3 | 1.6 |  |
| Tnfrsf18 | 2.4 | 1.5 | 2.4 | 1.5 | 0.9 | 1.5 |  |
| Ccdc47 | 3.1 | 2.0 | 3.1 | 2.0 | 2.3 | 2.3 |  |
| Mrpl32 | 1.3 | 2.1 | 2.1 | 1.3 | 1.0 | 1.3 |  |
| Chd9 | 0.8 | 2.1 | 2.1 | 1.3 | 1.0 | 1.3 |  |
| EG640050 /// Rps7 | 2.2 | 1.4 | 2.2 | 1.3 | 1.5 | 1.5 |  |
| Ctbs | 2.5 | 1.0 | 2.5 | 1.9 | 0.5 | 1.9 |  |
| Pitpnc1 | 2.0 | 2.2 | 2.2 | 1.5 | 1.7 | 1.7 |  |
| Csf2rb /// Csf2rb2 | 1.1 | 2.2 | 2.2 | 1.7 | 1.1 | 1.7 |  |
| Wasl | 2.4 | 1.3 | 2.4 | 1.9 | 0.7 | 1.9 |  |
| Mrpl55 | 2.0 | 1.3 | 2.0 | 1.5 | 1.0 | 1.5 |  |
| Hint2 | 2.0 | 1.2 | 2.0 | 1.6 | 1.6 | 1.6 |  |
| Ppa1 | 2.4 | 1.8 | 2.4 | 2.0 | 1.3 | 2.0 |  |
| Sfxn1 | 2.4 | 1.8 | 2.4 | 2.2 | 2.2 | 2.2 |  |
| Egln1 | 0.8 | 2.1 | 2.1 | 2.2 | 1.8 | 2.2 |  |
| Hnrpm | 1.7 | 2.0 | 2.0 | 1.9 | 2.3 | 2.3 |  |
| Ankrd11 /// LOC629623 | 2.0 | 0.6 | 2.0 | 2.3 | 2.4 | 2.4 |  |
| Nktr | 2.2 | 2.2 | 2.2 | 3.1 | 1.6 | 3.1 |  |
| Pick1 | 1.4 | 2.1 | 2.1 | 4.0 | 1.1 | 4.0 |  |

Genes with only 2-fold increase in expression, between vehicle and estrogen treatments are listed.

Transcripts regulated by ERα (genes up-regulated in the peri-ductal stroma of ERα(+/+) but not in ERα(-/-) mice).
